# The Role of Peroxisome Proliferator-Activated Receptors in Colorectal Cancer

**DOI:** 10.1155/2012/876418

**Published:** 2012-09-11

**Authors:** Joo-In Park, Jong-Young Kwak

**Affiliations:** Department of Biochemistry, College of Medicine, Dong-A University, 3 Ga 1, Dongdaesin-Dong, Seo-Gu, Busan 602-714, Republic of Korea

## Abstract

Colorectal cancer is one of the most common cancers in the world. Dietary fat intake is a major risk factor for colorectal cancer. Some nuclear hormone receptors play an important role in regulating nutrient metabolism and energy homeostasis. Among these receptors, special attention has been focused on the role of peroxisome proliferator-activated receptors (PPARs) in colorectal cancer, because PPARs are involved in regulation of lipid and carbohydrate metabolism. PPARs are ligand-activated intracellular transcription factors. The PPAR subfamily consists of three subtypes encoded by distinct genes named PPAR**α**, PPAR**β**/**δ**, and PPAR**γ**. PPAR**γ** is the most extensively studied subtype of PPARs. Even though many investigators have studied the expression and clinical implications of PPARs in colorectal cancer, there are still many controversies about the role of PPARs in colorectal cancer. In this paper, the recent progresses in understanding the role of PPARs in colorectal cancer are summarized.

## 1. Introduction

Colorectal cancer is one of the most common cancers in the world. Its incidence appears to be increasing, particularly in developed countries [1–3]. Colorectal carcinogenesis results from the loss of the normal regulatory pathways involved in cell proliferation and cell death. Especially, molecular alterations of multiple pathways including Wnt (Wingless type)/adenomatous polyposis coli (APC), cyclooxygenase-2 (COX-2), and Ras are known to play important roles in progression of colorectal cancer. Recent progresses in the development of new chemotherapeutic agents have improved the prognosis of colorectal cancer patients [4]. However, for most patients with advanced colorectal cancer, it is still difficult to achieve a complete remission, especially with surgery or chemotherapy. Therefore, significant effort has been exerted to identify novel drug targets for both the prevention and treatment of colorectal cancer.

**Figure 1:**
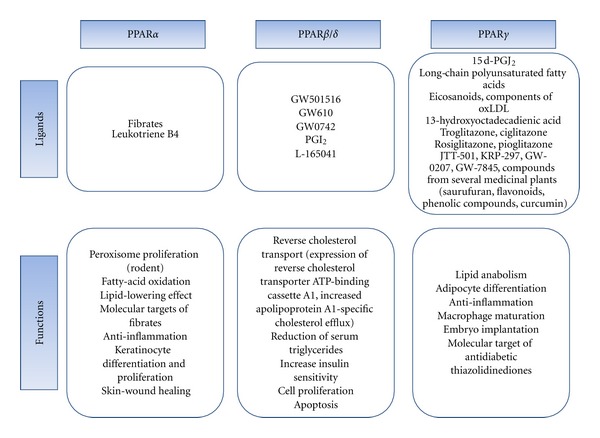
Summary of ligands and functions of each PPAR.

The peroxisome proliferator-activated receptors (PPARs) belong to members of the nuclear hormone receptor superfamily including receptors for steroid, retinoid, vitamin D, and thyroid hormones [5]. PPARs have received the attention of investigators interested in studying about the intracellular pathways that control signal transduction and gene transcription since their discovery in 1990. The name of PPARs was derived from its property to proliferate peroxisomes in rodent liver, where PPAR*α* plays the major role. However, none of the PPARs could be contributed to peroxisome proliferation in humans [6]. PPARs are metabolic regulators involved in the regulation of glucose and lipid homeostasis. Ligand-activated PPAR forms heterodimer with the retinoid X receptor (RXR) and binds to a PPAR response element (PPRE) to regulate the transcription of numerous target genes [7, 8]. The target genes are involved in cell differentiation, proliferation, immune/inflammation response, and lipid metabolism. PPAR subfamily consists of three members such as PPAR*α*, PPAR*β*/*δ*, and PPAR*γ*. PPAR isoforms consist of activation domain (A/B), DNA-binding domain (C), hinge region (D), and ligand-binding domain (E). Each subtype has different characteristics as summarized in Figure 1. PPAR*α* is expressed in brown adipose tissue, liver, kidney, heart, skeletal muscle, and enterocyte. Ligands for PPAR*α* are fibrates, leukotriene B4, and so on. PPAR*α* is involved in peroxisome proliferation, lipid catabolism, lipid-lowering effect, anti-inflammation, keratinocyte differentiation and proliferation, and skin wound healing. PPAR*β*/*δ* is ubiquitously expressed and is involved in reverse cholesterol transport, cell proliferation, apoptosis, and so on. PPAR*γ* is expressed in adipose tissue, colon, immune system, hematopoietic cells, and retina. PPAR*γ* is involved in lipid anabolism, adipocyte differentiation, control of inflammation, macrophage maturation, embryo implantation, and molecular targets of antidiabetic thiazolidinediones (Reviewed in [9]). Of the three PPARs identified to now, PPAR*γ* represents the most promising target in view of the many reports implicating this molecule in cancer cell growth.

## 2. The Role of PPAR*α* in Colorectal Cancer

 Although the procarcinogenic effects of PPAR*α* in rodent hepatocarcinoma are evident, less is known about the role of PPAR*α* in human colorectal cancer. Previous studies showed that activation of PPAR*α* by exogenous agonists causes inhibition of tumor cell growth in cell lines derived from colorectal cancer [10]. However, there is no evidence showing that PPAR*α* expression is elevated in human cancers. Recent studies have shown that aspirin and other nonsteroidal anti-inflammatory drugs reduce the relative risk of developing colorectal cancers [11, 12]. The products of COX activity are known to be involved in carcinogenesis [13–15]. COX-2 is not expressed in most normal tissues but is induced upon stimulation by inflammatory agents, and also by oncogenes, growth factors, carcinogens, and tumor promoters [16–21]. Overexpression of COX-2 contributes to colorectal carcinogenesis by promoting the invasiveness of malignant cells, inhibiting apoptosis, and supporting angiogenesis [22–24]. Furthermore, human colorectal carcinoma patients with COX-2 positive tumors show a significantly poorer prognosis than those with tumors negative for COX-2 [25]. It was recently demonstrated that bile acids, particularly secondary bile acids such as lithocholic acid and chenodeoxycholic acid, can stimulate cell proliferation [26] and act as tumor promoters in colon carcinogenesis [27, 28]. Previous reports have suggested that endogenous bile acids are ligands for nuclear receptors such as farnesoid X receptor (FXR), pregnane X receptor (PXR), and vitamin D receptor (VDR) [29–32]. A recent study reported that bile acids also induce the expression of the PPAR*α* gene via activation of FXR and leads to expression of COX-2 contributing to colorectal carcinogenesis [33]. These data suggest that PPAR*α* has the tumor-promoting activity.

There is a growing importance of chemotherapy for malignant colon cancers. However, resistance to anticancer drugs is still a major obstacle in the failure of chemotherapy in colorectal cancer patients. Tong et al. demonstrated that decreased expression of PPAR*α* confers resistance to hydroxycamptothecin, an inhibitor of topoisomerase I [34]. Thus, they suggest that increased expression of PPAR*α* is necessary to overcome hydroxycamptothecin resistance even though its reason is not clarified. 

## 3. The Role of PPAR*γ* in Colorectal Cancer 

The PPAR*γ* is a ligand-activated transcription factor of the nuclear receptor superfamily [35, 36] and is expressed in a variety of malignant tissues including prostate, breast, and colon [37–41]. Upon activation, PPAR*γ* forms heterodimer with RXR and mediate transcriptional activation by binding to the PPRE [7, 8]. In the inactive state, association of various corepressor molecules with PPAR*γ* (e.g., nuclear receptor corepressor or silencing mediator for retinoid receptor and thyroid hormone receptors) prevents this complex from binding to DNA. For transcriptional transactivation of PPAR*γ*, recruitment of coactivators (e.g., CCAAT/enhancer-binding protein, cyclic adenosine monophosphate response-element-binding protein, steroid receptor coactivator-1, receptor-interacting protein 140, PPAR*γ* coactivator-1, and PPAR*γ* binding protein) is required which replace co-repressors from the heterodimer complex. Transcriptional transrepression occurs through a genome independent mechanism and is mediated via physical association of the heterodimer with other activated transcription factors (STAT, NF-*κ*B, and AP-1) thereby blocking their functions (reviewed in [42]). PPAR*γ* has been known to be related to inflammation, immune response, and pathogenesis of some disorders including obesity, atherosclerosis, cancer, and so on [43]. There are natural ligands for PPAR*γ*, including long-chain polyunsaturated fatty acids, eicosanoids, components of oxidized low density lipoproteins (oxLDL) [44], and oxidized alkyl phospholipids. The prostaglandin J_2_ derivative, 15d-PGJ_2_ is the most potent endogenous ligand for the PPAR*γ* receptor. The antidiabetic thiazolidinedione (TZD) class of drugs including troglitazone, rosiglitazone, pioglitazone and ciglitazone are synthetic ligands for PPAR*γ* [44]. Recent studies have focused on the effect of PPAR*γ* ligands as anticancer agents. However, there are still controversies about the antitumor activity of PPAR*γ* agonists. Thus, this paper describes the role of PPAR*γ* in colorectal cancer and its detailed mechanisms clarified until now. 

### 3.1. The Role of PPAR*γ* as a Tumor Suppressor in Colorectal Cancer

Several studies have focused on the putative association between the various polymorphisms and mutations of the PPAR*γ* gene and the occurrence of cancer. It was described that 4 somatic PPAR*γ* gene mutations resulting in reducing its function occurred in 55 sporadic colon cancers [45]. However, Ikezoe et al. [46] analyzed 397 clinical samples and cell lines including colon, breast, and lung cancers for mutations of PPAR*γ* gene and showed the absence of PPAR*γ* gene mutations. These data suggest that PPAR*γ* mutations may occur in cancers but they are rare. 

**Figure 2:**
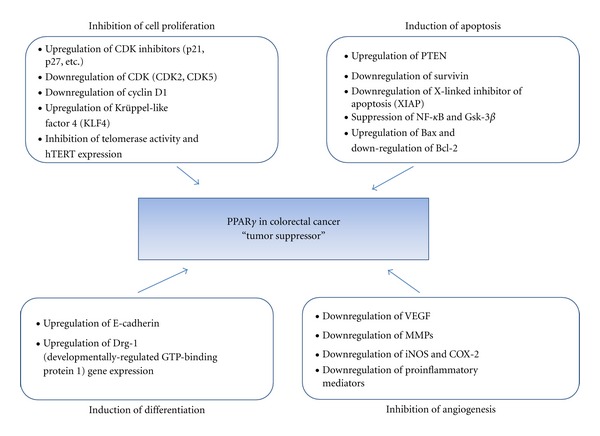
Potential molecular mechanisms for PPAR*γ* as tumor suppressor in colorectal cancer.

There has been substantial accumulation of experimental data supporting that synthetic PPAR*γ* ligands as well as 15d-PGJ_2_ induce apoptosis in several types of cancer cells [41, 43]. Although increasing evidence has established that PPAR*γ* agonist induces growth arrest in cancer cells, the molecular mechanism of the growth inhibition by PPAR*γ* agonist is not well understood and complicated. This paper describes some of the molecular mechanisms for anticancer activity of PPAR*γ* (Table 1 and Figure 2) as follows. 

#### 3.1.1. Inhibition of Cell Proliferation and Induction of ****Apoptosis

**(1) Upregulation of PTEN**

The Phosphatase and Tensin Homolog (PTEN) tumor suppressor gene modulates several cellular functions, including cell migration, survival, and proliferation by inhibiting phosphatidylinositol 3-kinase (PI-3K)-mediated signaling cascades [74]. Previous studies have demonstrated that rosiglitazone, a synthetic ligand for PPAR*γ*, upregulates PTEN expression in Caco2 colorectal cancer cells [47]. Dai et al. also show that treatment of colon cancer cells with rosiglitazone stimulates expression of tumor suppressor gene PTEN. This effect is probably mediated through the binding of PPAR*γ* on PPRE in the promoter of PTEN [48]. Inhibition of the PI-3K/Akt pathway by increased PTEN expression is believed to underlie this effect of the PPAR*γ* ligand.

**(2) Downregulation of Survivin**

Survivin is one of the inhibitors of apoptosis protein (IAP) family since it is overexpressed in almost every human tumor that has been studied, but is barely detectable in most normal adult tissues [75]. Overexpression of survivin is associated with poor clinical outcome with reduced tumor cell apoptosis in patients with colorectal cancer [76, 77]. PPAR*γ* agonist GW7845 induced cell death through downregulation of survivin in colorectal cancer cells [49].

**(3) Downregulation of X-Linked Inhibitor of Apoptosis (XIAP)**

XIAP can inhibit apoptosis by binding and thereby inactivating caspases including caspase-9 and the effector caspases (-3 and -7) [78]. Qiao et al. showed that 15d-PGJ_2_ and troglitazone mediate XIAP downregulation in colon cancer cells by facilitating ubiquitination and proteasomal degradation [50]. In addition, Lee et al. demonstrated that pioglitazone induces apoptosis through downregulation of XIAP via unknown mechanism in colorectal cancer cell lines [51].

**(4) Suppression of NF-*κ*B and GSK-3*β***

The transcription factor NF-*κ*B is involved in the regulation of various genes, including metalloproteinases (MMPs), inflammatory response genes, and a number of antiapoptotic genes including cIAP1, cIAP2, and glycogen synthase kinase-3 (GSK-3) [79]. Its activation is also associated with cell proliferation, cell cycle progression, promotion of tumor growth, angiogenesis, and metastasis through the expression of genes participating in malignant conversion and tumor promotion [80–82]. Ban et al. showed that PPAR*γ* agonist, troglitazone inhibits colon cancer cell growth via inactivation of NF-*κ*B by suppressing GSK-3*β* activity [52].

**(5) Upregulation of Cyclin-Dependent Kinase (CDK) Inhibitors, Downregulation of CDK and Downregulation of Cyclin D1**

Interestingly, CDK5 protein expression and kinase activity were significantly inhibited by ciglitazone, which was associated with ciglitazone-induced antiproliferation in colon cancer HT-29 cells [53]. Cyclin D1 is involved in G1/S progression and increased proliferation. PPAR*γ* activation in intestinal epithelial cells results in the inhibition of cell cycle and S-phase entry though a decrease in cyclin D1 expression [54, 55]. PPAR*γ* ligand treatment not only decreases the protein level of cyclin D1, but also increases the CDK inhibitors p21^CIP^ and p27^KIP1^ through both increased transcriptional activity and inhibition of proteasome degradation in colorectal cancer cells [56, 57]. Ciglitazone also inhibited G1/S cell cycle progression through upregulation of p27 and inhibition of Cdk2 activity in HT-29 cells [56]. Fajas et al. [83] suggested that PPAR*γ* activation in the presence of RB results in G1 arrest, whereas in the absence of RB, cells accumulate in G2/M, endoduplicate, and undergo apoptosis. Lee et al. [51] also showed that pioglitazone treatment leads to G2/M block through downregulation of cyclin B1 and cdc2 and upregulation of p21 in RB-deficient human colorectal cancer SNU-C4 and SNU-C2A cells. Thus, these studies suggest that the antiproliferative or proapoptotic effects of PPAR*γ* agonist are associated with its ability to regulate the expression of various genes which are involved in controlling the cell cycle and cell survival/death.

**(6) Downregulation of COX-2**

Most of the current studies showed that COX-2 contributes to tumorigenesis through various mechanisms and overexpression of COX-2 can stimulate tumor growth, invasion, and metastasis [84, 85]. A previous study showed that pioglitazone induces apoptosis through the downregulation of COX-2, activation of caspase-3, downregulation of Bcl-2 and upregulation of Bax in RB-deficient human colorectal cancer cells [51].

**(7) Upregulation of Krüppel-Like Factor 4 (KLF4)**

KLF4 is a member of the Krüppel-like zinc finger transcription factor family. It is extensively expressed in the epithelial cells of the gastrointestinal tract [86–88]. Over-expression of KLF4 in colon cancer cells caused inhibition of DNA synthesis and cell growth [89, 90]. Zhi and Tseng demonstrated that 15d-PGJ_2_ inhibits proliferation of HT-29 human colon cancer cells and induces upregulation of KLF4 mRNA and protein through the activation of MEK/ERK and STAT-dependent pathway [58]. They provided a novel mechanism for the antitumorigenic actions of 15d-PGJ_2_. In addition, rosiglitazone treatment of colorectal cancer cells caused to G1 arrest because increased expression of KLF4 by rosiglitazone leads to increased expression of p21 and decreased expression of cyclin D1 [59]. These data suggest that KLF4 is a nodal player in a network of PPAR*γ*-regulated genes.

**(8) Upregulation of Bax and Downregulation of Bcl-2**

In colon cancer cells, treatment of the PPAR*γ* ligands (pioglitazone, troglitazone) induces apoptosis through upregulation of the proapoptotic protein Bax and downregulation of the antiapoptpotic protein Bcl-2 [51, 60, 61]. Alternative expression of Bax and Bcl-2 causes apoptosis by the release of cytochrome c and subsequent activation of several effector caspases.

**(9) Inhibition of Telomerase Activity and hTERT Expression through Modulation of the Myc/Mad/Max Network**

The telomerase stabilizes telomere length by adding TTAGGG repeats to telomeres [91, 92]. Telomerase activity has been detected in almost all human tumors [93, 94] but not in adjacent normal cells [95, 96]. Human telomerase is composed of human telomerase RNA, telomerase-associated protein 1 and human telomerase reverse transcriptase (hTERT) [91, 97]. The forced expression of hTERT in normal human cells has been reported to increase their lifespan [98], while the expression of dominant-negative hTERT in human cancer cells has been known to inhibit telomerase and cause telomere shortening [99, 100]. A recent study shows that 15d-PGJ_2_ and rosiglitazone inhibit Caco-2 colon cancer cell proliferation through the inhibition of telomerase activity and hTERT expression. In addition, it was demonstrated that the inhibition of hTERT expression in Caco-2 cells depends on the downregulation of c-Myc and the upregulation of Mad 1 by PPAR*γ* ligands [62].

#### 3.1.2. Induction of Cellular Differentiation

 PPAR*γ* has been demonstrated to induce differentiation in solid tumors both *in vitro* and *in vivo *[101]. In colon cancer cells, activation of PPAR*γ* by troglitazone treatment inhibits growth and metastasis through differentiation-promoting effects, such as the marked increase in p21 Waf-1, developmentally regulated GTP-binding protein 1 (DRG-1), and E-cadherin in human colon cancer cells [63]. These effects involve modulation of the E-cadherin/*β*-catenin system and upregulation of Drg-1 gene expression. 

#### 3.1.3. Inhibition of Angiogenesis

Angiogenesis, a formation of new capillaries from the preexisting vessels, is a complex process involved in the degradation of the basement membrane by cellular proteases, the penetration and migration of endothelial cells into the extracellular matrix, endothelial cell proliferation, tube formation, and vessel stabilization [102]. Inhibition of angiogenesis may contribute to the mechanism by which PPAR*γ* agonists halt the cancer process. Several studies demonstrated that PPAR*γ* agonist inhibits angiogenesis through the following mechanisms.

**(1) Downregulation of Vascular Endothelial Growth Factor (VEGF)**

VEGF is involved in angiogenesis [103, 104]. VEGF expression is increased in several cancers including colorectal and other tumors [105, 106]. It was shown that rosiglitazone inhibited angiogenesis via the downregulation of VEGF and VEGF mRNA in pancreatic cancer xenografts [64].

**(2) Downregulation of Matrix Metalloproteinases (MMPs)**

The process of cancer cell invasion is dependent on the degradation of the extracellular matrix (ECM) by MMPs. MMPs are a family of proteases cleaving several macromolecules of the ECM [107]. 15d-PGJ_2_ has been reported to have inhibitory effects on the proliferation and invasiveness of colon cancer cell lines which are associated with G1 cell cycle arrest and downregulation of MMP-7 synthesis [65].

**(3) Downregulation of iNOS and COX-2**

It has been shown that both COX-2 and inducible nitric oxide synthase (iNOS) are overexpressed in various human cancers [108]. It was reported that iNOS is associated with altered expression of important modulators of angiogenesis [108]. 15d-PGJ_2_ downregulates iNOS [66–68] and COX-2 [69–71]. The expression of COX-2 and iNOS is regulated by NF-*κ*B. The recent several studies have demonstrated that 15d-PGJ_2_ can act as a negative regulator of proinflammatory signaling through blocking the NF-*κ*B activation pathway at multiple levels via covalent modification of NF-*κ*B or its regulators [72]. Thus, antiangiogenic effects of 15d-PGJ_2_ might be associated with disruption of NF-*κ*B and subsequent blockade of iNOS and COX-2 expression.

**(4) Downregulation of Proinflammatory Mediators**

The potential mechanism of angiogenesis inhibition by 15d-PGJ_2_ may involve downregulation of pro-inflammatory mediators. Both physiological and pathological angiogenesis can be stimulated by pro-inflammatory cytokines, such as IL-1 and TNF-*α*. Certain cytokines (e.g., IL-6 and CSF-1) can influence the phenotype and the function of tumor-associated macrophages and indirectly stimulate tumor invasiveness and angiogenesis [109]. Tumor-associated macrophages play an important role in tumor progression due to production of several angiogenic factors, such as VEGF, IL-8, inflammatory cytokines (IL-1 and IL-10) and proteases (MMP-2 and MMP-9) [109]. Thus, 15d-PGJ_2_ inhibits angiogenesis through suppression of such pro-inflammatory cytokines [73]. Induction of several pro-inflammatory cytokines, such as TNF-*α*, IL-1, and IL-8, is regulated at the transcription level by NF-*κ*B. It is still unclear whether 15d-PGJ_2_ exerts an anti-angiogenic effect through inhibition of NF-*κ*B-dependent induction of pro-inflammatory mediators or through downregulation of cancer cell-derived pro-inflammatory cytokine release which is NF-*κ*B-independent. Hence, further investigations are necessary to clarify the signaling pathways that delineate the anti-angiogenic effects of 15d-PGJ_2_.

### 3.2. The Role of PPAR*γ* as a Tumor Promoter in Colorectal Cancer

**Figure 3:**
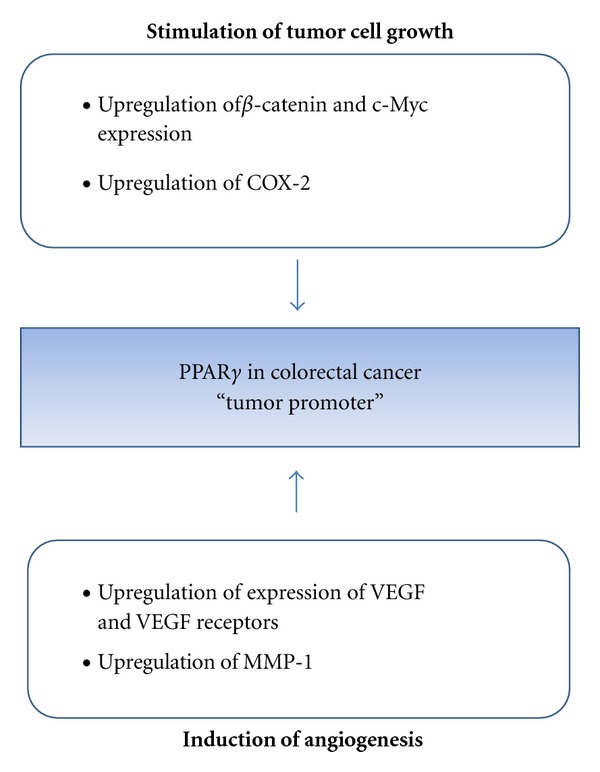
Potential molecular mechanisms for PPAR*γ* as tumor promoter in colorectal cancer.

In contrast to above described, PPAR*γ* has been known to have procarcinogenic activity such as stimulation of tumor cell growth and induction of angiogenesis. This review describes some mechanisms for it as summarized in Table 2 and Figure 3.

#### 3.2.1. Stimulation of Tumor Cell Growth

Although the majority of publications indicate that PPAR*γ* agonists have potent antiproliferative properties in several types of cancer cells, there are some reports demonstrating the cell growth promoting effects of 15d-PGJ_2_ and other PPAR*γ* ligands. It was shown that activation of PPAR*γ* by troglitazone increased the frequency and the size of colon tumors in C57BL/6J-APC^Min/+^ mice [116, 117]. In addition, a recent study shows that low concentration of 15d-PGJ_2_ and pioglitazone can promote the growth of APC-mutated HT-29 colon cancer cells *in vitro* and *in vivo *[110].

**(1) Upregulation of *β*-Catenin and c-Myc Expression**

The Wnt/*β*-catenin pathway plays a critical role in the development of colon cancer [118]. Choi et al. showed that low concentrations of 15d-PGJ_2_ and pioglitazone promote the HT-29 colon cancer cells *in vitro* and *in vivo* through increase in *β*-catenin and c-Myc expression [110].

**(2) Upregulation of COX-2**

As previously mentioned, 15d-PGJ_2_ is one of the major final products of COX-2. Since abnormal overexpression of COX-2 was observed in several cancer cells, COX-2 has been shown to contribute to carcinogenesis by promoting cell proliferation and angiogenesis as well as by protecting cells from apoptosis [119]. The regulation of COX-2 synthesis occurs mainly at the transcriptional level, although mRNA stabilization is also involved. A recent study has shown that 15d-PGJ_2_ enhances COX-2 expression through ROS-Akt-driven AP-1 activation in human breast cancer cells [111].

#### 3.2.2. Induction of Angiogenesis

It has been reported that PPAR*γ* agonist can induce angiogenesis in various cell lines. Several studies provided some molecular mechanisms for induction of angiogenesis by PPAR*γ* agonist. Here, this paper summarizes the potential molecular mechanisms for enhanced metastasis and invasion by PPAR*γ* agonist clarified until now.

**(1) Upregulation of Expression of VEGF and VEGF Receptors**

It was shown that the mRNA expression of VEGF was augmented by 15d-PGJ_2_ and troglitazone in vascular smooth muscle cell, human monocytes/macrophages, human colorectal cancer cells and human coronary artery endothelial cells [112, 113]. More recently, 15d-PGJ_2_ and troglitazone have been reported to increase the expression of VEGF and its receptors (Flt-1 and KDR) in myofibroblasts [114].

**(2) Upregulation of MMP-1**

Kim et al. reported that 15d-PGJ_2_ enhances the angiogenesis by upregulation of MMP-1 [115]. MMP-1 is a major proteinase degrading native fibrillar collagens. MMP-1 is produced by a variety of cell types, including endothelium. It is implicated in several pathological processes such as tumor invasion and restenosis [120]. In addition, Kim et al. suggested that iron may contribute to increased metastasis and invasiveness by 15d-PGJ_2_ in human breast cancer cells [115]. Thus, these studies suggest the regulation of MMP-1 expression by 15d-PGJ_2_ may be more complex than expected.

## 4. The Role of PPAR*β*/*δ* in Colorectal Cancer

PPAR*β*/*δ* is also expressed in the colon and can be activated by fatty acids. In recent studies, it was shown that PPAR*β*/*δ* plays a central role in the differentiation of Paneth cells and innate immunity [121]. The role of PPAR*β*/*δ* in colorectal cancer is more controversial than that of PPAR*γ*. Recent studies have shown that PPAR*β*/*δ* is involved in the pathogenesis of colorectal cancer [122]. Inactivation of APC upregulates PPAR*β*/*δ* expression in colorectal cancer cells [122]. It has also been reported that PPAR*β*/*δ* levels increase in colorectal tumor after treatment with the potent carcinogen azoxymethane (AOM) [123]. The increased expression of PPAR*β*/*δ* could potentially be activated by endogenous ligands such as COX-derived prostacyclin [123]. It was proposed that PPAR*β*/*δ* activation would initiate the expression of target genes, which still remain to be identified, and enhance cell growth. In support of this model, PPAR*β*/*δ*-null HCT116 cells have reduced tumorigenecity in a xenograft model [124]. PPAR*β*/*δ* expression levels in colorectal cancers are higher than in normal mucosa, supporting the hypothesis that APC suppresses activity of *β*-catenin/Tcf-4 transcription of target genes, including PPAR*β*/*δ*, c-myc, and cyclin D1 [122, 123]. PPAR*β*/*δ* expression and activity are also induced by oncogenic *K-ras* in rat intestinal epithelial cells [125]. These studies support a procarcinogenic role of PPAR*β*/*δ* in colorectal cancer. 

A few mechanisms have been proposed to explain the procarcinogenic effect of PPAR*β*/*δ*. Di-Poï et al. suggested that PPAR*β*/*δ* activation increases the expression of 3-phosphoinositide-dependent-protein kinae 1 (PDPK1) and integrin-linked kinase (ILK), and decreases the expression of PTEN, causing increased phosphorylation of AKT, leading to antiapoptotic signaling and enhanced cell survival [126]. Another related mechanism is derived from the observation that ligand activation of PPAR*β*/*δ* increases the expression of VEGF through a PPAR*β*/*δ*-dependent mechanism, causing increased phosphorylation of AKT, which promotes cell survival by blocking apoptosis [127]. In addition, Kwak et al. [128] demonstrated that PPAR*β*/*δ*-binding aptamers suppressed transcription from natural promoters of VEGF-A and COX-2 and inhibited tumorigenic potential of colon-cancer cells. These data suggest that PPAR*β*/*δ* play an important role in transcription of tumor-promoting genes such as VEGF-A and COX-2. 

 However, other studies conflict with those reports. Targeted deletion of APC alleles reduces PPAR*β*/*δ* expression in mouse intestine [129]. PPAR*β*/*δ* expression in human colorectal cancers or intestinal polyps of APC^Min/+^ mice are either unchanged or downregulated as compared with normal controls (reviewed in [130, 131]).

The conflicting results about the effect of PPAR*β*/*δ* on intestinal tumorigenesis in APC^Min/+^- and AOM-treated mice may be related to differences in the specific targeting strategy employed to delete PPAR*β*/*δ* [127]. Deletion of PPAR*β*/*δ* exon 4 and/or 5, which encodes an essential portion of the DNA-binding domain, is thought to disrupt PPAR*β*/*δ* function as a nuclear transcriptional factor and to inhibit colonic carcinogenesis [127, 132]. Increased expression of VEGF in colon tumors was suppressed by loss of PPAR*β*/*δ* expression [133]. These findings indicate that PPAR*β*/*δ* has an important role in promoting colonic tumorigenesis. The deletion of exon 8 [134, 135], the last PPAR*β*/*δ* exon, is postulated to generate a hypomorphic PPAR*β*/*δ* protein that remains at least partly functional. 

In a mouse mammary tumor model, treatment with the PPAR*β*/*δ* agonist GW501516 accelerated tumor formation, while a PPAR*γ* agonist GW7845 delayed tumor growth [136]. This observation suggests that there are distinct mechanistic differences between PPAR*γ* and PPAR*β*/*δ* in regulating tumor progression. A recent study showed that PPAR*β*/*δ* confers resistance to PPAR*γ*-induced apoptosis by increasing the expression of survivin [49]. 

 Recently, Yang et al. [137] showed that the specific knockdown of PPAR*β*/*δ* in colon-cancer cell lines results in more malignant morphologies, larger colonies and less CEA production, and enhances cell-fibronectin adhesion, without effects on cell invasion and migration. These findings indicate that PPAR*β*/*δ* may facilitate differentiation and inhibit the cell-fibronectin adhesion of colon cancer, having a protective role in the carcinogenesis and progression of colon cancer. Further immunohistochemistry data reveal that the expression of PPAR*β*/*δ* is closely associated with the differentiation and tumor-node-metastasis stage of rectal cancer. It was also shown that PGI_2_ and L-165041, a synthetic PPAR*δ* ligand, activate PPAR*δ* and upregulate PPAR*δ*-mediated 14-3-3*ε* expression. 14-3-3*ε* binds and sequesters Bad in cytosol. PGI_2_-induced 14-3-3*ε* upregulation is accompanied by augmented Bad sequestration and protects HT-29 cells from Bad-triggered mitochondrial leakage of proapoptotic factors and the consequent apoptosis [138]. 

## 5. Conclusion and Future Directions

 Even though the extensive studies to clarify the role of PPARs in colorectal cancer using several PPAR agonists and gene knockout experiments were performed, there are still many controversies about them. PPAR ligands induce many physiological changes, including increased oxidation of fatty acids, which contributes to decreasing serum lipids and reducing body weight; and inhibition of inflammatory signaling. There are good reasons to suggest that PPAR agonists should be potential candidates for treating and preventing colorectal cancer, because obesity and chronic inflammation are major risk factors for colorectal cancer. It is interesting to note that there is an overlap in target genes regulated by each PPAR, but the physiological effects induced by selective PPAR agonists are unique owing to the complexity of the PPAR-dependent and the PPAR-independent effects that each agonist induces. To completely understand the role of PPARs in colorectal cancer, it is necessary to dissect the complex regulation of PPAR expression and to examine interactions of each PPAR with other nuclear receptors and signalling molecules involved in cell proliferation and cell death in the near future.

## Figures and Tables

**Table 1:** Potential molecular mechanisms for anticancer activity of PPAR*γ*.

Actions and molecular mechanisms	References
(1) Inhibition of cell proliferation and induction of apoptosis	
(1) Upregulation of PTEN	[47, 48]
(2) Downregulation of survivin	[49]
(3) Downregulation of XIAP	[50, 51]
(4) Suppression of NF-*κ*B	[52]
(5) Up-regulation of cyclin-dependent kinase (CDK) inhibitors, down-regulation of CDK, and down-regulation of cyclin D1	[51, 53–57]
(6) Down-regulation of COX-2	[51]
(7) Up-regulation of Krüppel-like Factor4 (KLF4)	[58, 59]
(8) Up-regulation of Bax and down-regulation of Bcl-2	[51, 60, 61]
(9) Inhibition of telomerase activity and hTERT expression	[62]
(2) Induction of cellular differentiation	
Up-regulation of E-cadherin and Drg-1 gene expression	[63]
(3) Inhibition of angiogenesis	
(1) Down-regulation of vascular endothelial growth factor (VEGF)	[64]
(2) Down-regulation of matrix metalloproteinases (MMP)	[65]
(3) Down-regulation of iNOS and COX-2	[66–72]
(4) Down-regulation of proinflammatory mediators	[73]

**Table 2:** Potential molecular mechanisms for procarcinogenic activity of PPAR*γ*.

Actions and molecular mechanisms	References
(1) Stimulation of tumor cell growth	
(1) Up-regulation of *β*-catenin and c-Myc expression	[110]
(2) Up-regulation of COX-2	[111]
(2) Induction of angiogenesis	
(1) Up-regulation of VEGF and VEGF receptor	[112–114]
(2) Up-regulation of MMP-1	[115]
